# Comparison Between Performances of In_2_O_3_ and In_2_TiO_5_-Based EIS Biosensors Using Post Plasma CF_4_ Treatment Applied in Glucose and Urea Sensing

**DOI:** 10.1038/s41598-019-39012-9

**Published:** 2019-02-28

**Authors:** Chun Fu Lin, Chyuan Haur Kao, Chan Yu Lin, Chia Shao Liu, Yi Wen Liu

**Affiliations:** 1 Department of Electronic Engineering, Chang Gung University, Taoyuan, Taiwan; 2 Kidney Research Center, Department of Nephrology, Chang Gung Memorial Hospital, Chang Gung University, Taoyuan, Taiwan; 3 Department of Electronic Engineering, Ming Chi University of Technology, New Taipei City, Taiwan

## Abstract

In this study, the effect of post-deposition tetrafluoromethane (CF_4_) plasma treatment on the physical and electrical characteristics of an In_2_TiO_5_ based electrolyte-insulator-semiconductor (EIS) sensor was investigated. Post-deposition CF_4_ plasma treatment typically improved the crystalline structure and repaired dangling bonds at the grain boundaries. We used the newly fabricated device to detect several ions, such as sodium and potassium, which are essential for many biological processes. The as-deposited and CF_4_ plasma treated In_2_TiO_5_ sensing window with an EIS structure was also able to detect the pH of a solution, different alkali ions (Na^+^ and K^+^), glucose, and urea. The sensing membrane after a 60-sec CF_4_ plasma treatment displayed improved biosensing characteristics, such as higher sensitivity (59.64 mV/pH), better drift rate, and a smaller hysteresis voltage of approximately 0.424 mV/h. The In_2_TiO_5_ sensing membrane treated with CF_4_ plasma is a promising material for use in EIS biosensing applications.

## Introduction

Over the last few decades, major advances have been made in the field of biosensors for the monitoring and control of many biochemical activities. Biosensors are extensively used for monitoring food and soil quality, as well as human biochemistry and other medical applications^1^. Ion-sensitive field-effect transistors (ISFETs) are considered excellent transducers for sensing biochemical reactions due to their small size, fast response, and reliability. Electrolyte-insulator-semiconductor (EIS) materials operate with the same working principle as ISFETs, but their fabrication process is relatively simple and inexpensive as compared to that of ISFETs^2^. The surface potential of the exposed insulator sensing area changes in response to the H^+^ concentration of the solution^3^. Therefore, selecting a proper insulating material with suitable characteristics is important to achieve high sensitivity and long-term reliability of the device.

Several metal oxides like indium-gallium-zinc oxide (InGaZnO, IGZO), zinc oxide (ZnO), and indium tin oxide (InSnO, ITO) have been developed as sensor membranes owing to their excellent sensing characteristics^4–6^. Indium oxide (In_2_O_3_) thin films have attracted significant attention as a sensing membrane because of its higher mobility (160 cm^2^/(V·s))^7^, higher melting point (1910 °C), larger band gap (3.5–3.7 eV)^8^, and insolubility in water. In_2_O_3_ films can easily be reduced to create an oxygen deficiency, where insufficient oxygen atoms are contained in the crystal structure^9^. This results in the formation of a nonstoichiometric In_2_O_3−x_ film with an increased number of defects. Thus, the main free carriers in In_2_O_3−x_ are the internal oxygen vacancies of the film, which also affect the chemical stability of the film. As a potential solution, CF_4_ plasma treatment of the In_2_O_3_ and In_2_TiO_5_ film can enhance the In-O bonding. In_2_O_3_ is widely used for gas sensing^10^, transparent conducting oxides^11^, and thin film transistors^12^ because of its excellent material characteristics.

The performance of a biosensor is conferred by doping with a transition metal, such as titanium (Ti). Ti affects the sensing membrane through the passivation of the defects in the bulk to the sensing performance and production of dangling bonds at the oxide interface^13,14^. Ti internalization also enhances device capacitance and reduces the reactivity with the surrounding moisture^15,16^.

Recent evidence demonstrated CF_4_ plasma treatment that improves the sensing performance of oxide materials for biosensing applications^17^. Fluorine incorporation improves the dielectric properties of the material by fixing the dangling bonds and replacing the weak bonds at the grain boundaries^18^. Fluorine introduces a net negative surface charge owing to its high electronegativity and improves the ability to capture positively charged H^+^ ions^19^. Moreover, pre- and post-deposition CF_4_ plasma treatment reduces metal-silicate formation at the oxide/silicon (Si) interface^20^.

**Figure 1:**
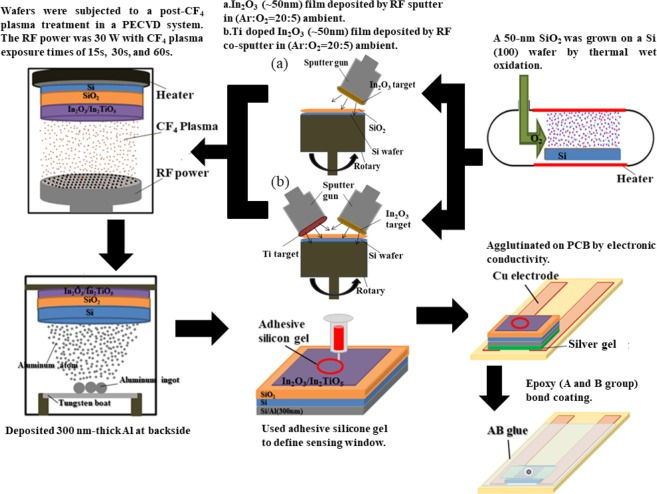
Process flow for the fabrication of In_2_O_3_ and In_2_TiO_5_ EIS with CF_4_ plasma treatment.

Here, we used a post-deposition CF_4_ plasma treatment on In_2_O_3_ and In_2_TiO_5_ thin films to improve their electrical and physical characteristics. We describe the consequence of the post-deposition CF_4_ plasma treatment on the sensing and physical characteristics of a deposited In_2_TiO_5_ thin film as a sensing membrane. The detailed process flow of the EIS structure fabrication is shown in Fig. 1. After fabricating the device, sensing characteristics including sensitivity, hysteresis, and drift of the device were measured. To determine the physical characteristics, X-ray diffraction (XRD) atomic force microscopy (AFM), and secondary ion mass spectroscopy (SIMS) analyses were done.

## Results and Discussion

### Physical characteristics

**Figure 2:**
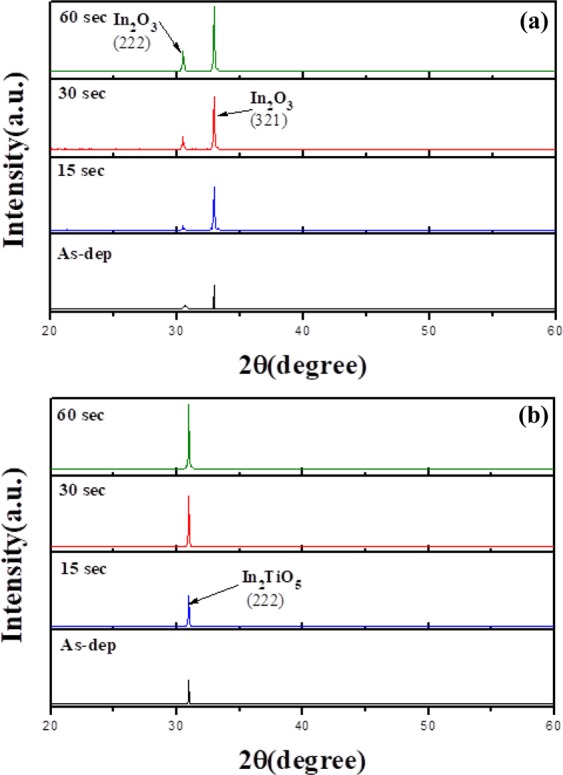
XRD spectra of the (**a**) In_2_O_3_ and (**b**) In_2_TiO_5_ membranes with CF_4_ plasma treatment for 15, 30, and 60 sec.

XRD was used to probe the crystalline structure of the materials. Figure 2(a,b) depicts XRD patterns of as-deposited and post-deposition CF_4_ plasma treated In_2_O_3_ and In_2_TiO_5_ membranes, respectively. The diffraction angles (2θ) ranged from 20 to 60°. All the In_2_O_3_ membrane samples displayed a cubic crystalline phase with two diffraction peaks, (222) and (321), at a diffraction angle of 30.5° and 33.10°, respectively^21^. A strong In_2_O_3_ (321) peak was observed for the sample annealed at 60 sec. In_2_TiO_5_ displayed a strong peak at (222) at a 2θ of 30.5. The major phase changed from (321) to (222) in XRD pattern of In_2_TiO_5_. The intensities of these three peaks were enhanced by the CF_4_ plasma treatment and further increased by increasing the plasma treatment time from 15 to 60 sec due to the improvement in the crystalline structure. The crystallinity improvement arises from the formation of a stronger fluorinated bond between indium and oxygen^18^. Therefore, after CF_4_ plasma treatment for 60 sec the In_2_O_3_ and In_2_TiO_5_ membranes would display higher intensity peaks than those prepared under other conditions. The XRD data indicated that internalization of Ti upgrades the thin film stoichiometry because it minimizes the number of dangling bonds on the dielectric surface and improves the temperature stability compared with In_2_O_3_ thin film^22,23^.

**Figure 3:**
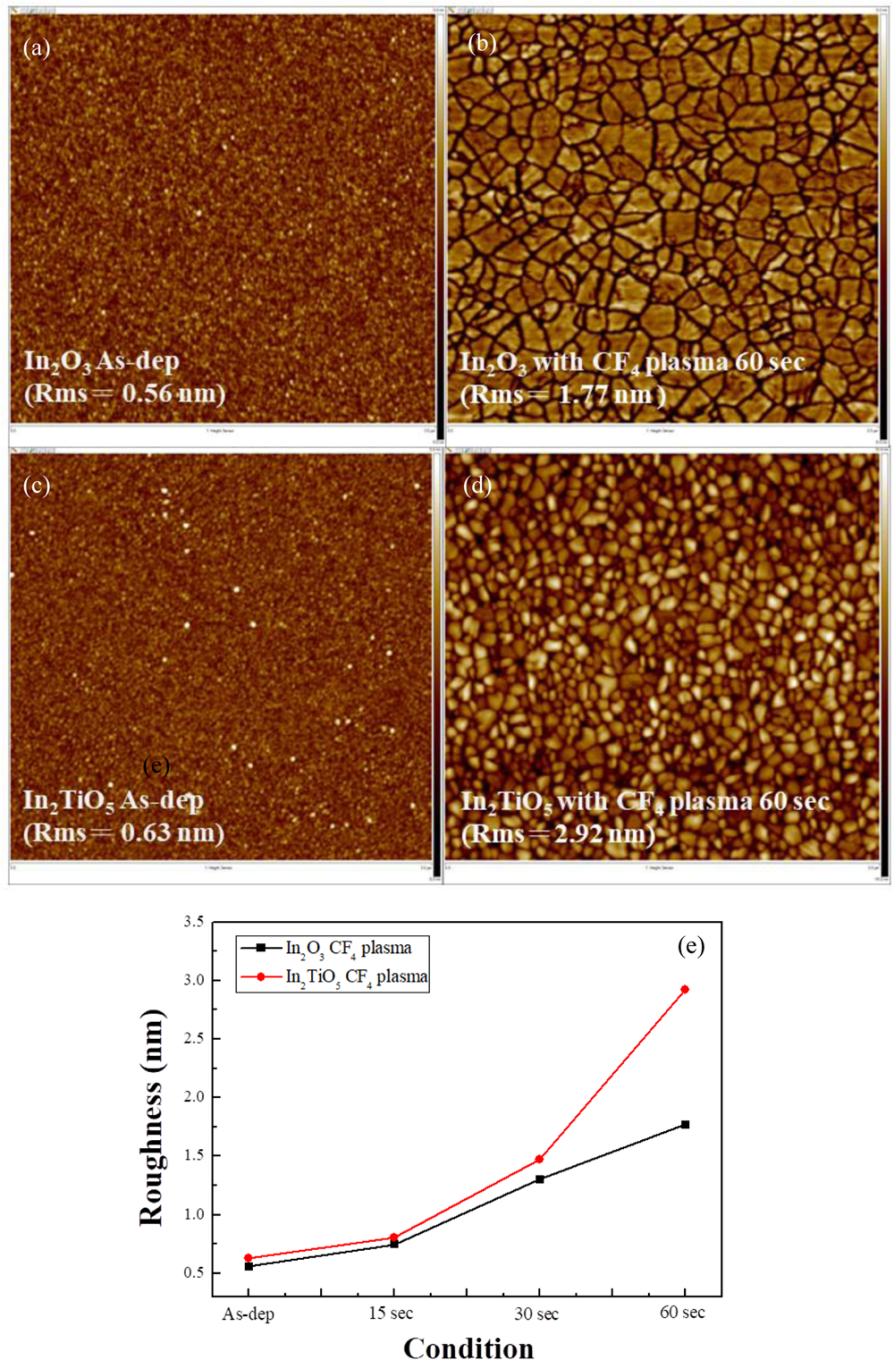
AFM of the surface of the (**a**) as-deposited In_2_O_3_ sample (R_rms_ = 0.557 nm), (**b**) In_2_O_3_ sample CF_4_ plasma treated for 60 sec (R_rms_ = 1.77 nm), (**c**) as-deposited In_2_TiO_5_ sample (R_rms_ = 0.628 nm), (**d**) In_2_TiO_5_ sample CF_4_ plasma treated for 60 sec (R_rms_ = 2.92 nm), and (**e**) AFM of the sensing membrane with CF_4_ plasma treatment for various times.

AFM was used to study the surface morphology of the devices treated with post-deposition plasma. Figure 3(a–d) show two-dimensional (2D) AFM images of the as-deposited and post-deposition CF_4_ plasma treated In_2_O_3_ and In_2_TiO_5_ samples. The AFM images of as-deposited In_2_O_3_ and In_2_TiO_5_ membranes revealed surface roughness of 0.557 nm and 0.628 nm, respectively. The roughness increased with In_2_O_3_ and In_2_TiO_5_ samples with increased CF_4_ plasma treatment time from 15 to 60 sec, as shown in Fig. 3(e). Samples doped with Ti achieve better surface roughness compared to bare In_2_O_3_. Ti has a higher affinity toward oxygen, and thus provides a larger grain size. This produces a rougher surface^23,24^. CF_4_ plasma treatment increases the interior grain size and causes plasma-induced morphological changes, which increase surface roughness and the number of surface sites, which in turn increase the sensitivity and linearity^25^.

**Figure 4:**
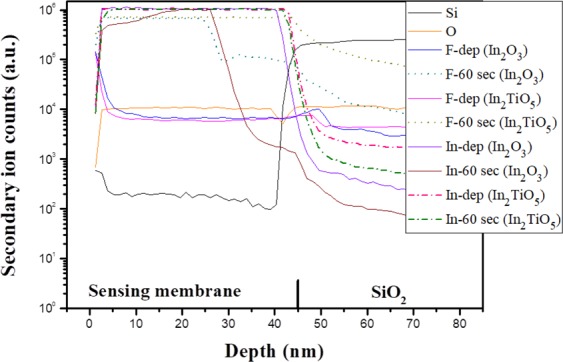
In_2_O_3_ and In_2_TiO_5_ sensing membrane SIMS profiles of CF_4_ plasma treated samples.

Secondary ion mass spectrometry analysis data of the In_2_O_3_ and In_2_TiO_5_ thin films with and without CF_4_ plasma treatment are shown in Fig. 4. The as-deposited sample displayed no trace of fluorine atoms. For all the samples with CF_4_ plasma treatment, fluorine atoms were evenly distributed throughout the entire oxide bulk areas. Fluorine content increased as the CF_4_ plasma treatment time increased from 15 to 60 sec. Ti-doped In_2_O_3_ membranes have less excess etching of the sensing membrane. Fluorine incorporation reduces interfacial defect states at the oxide/silicon interface and increases the overall quality of the deposited oxide layer^26^.

### Sensing characterization

The most important parameter of EIS structure is its flat-band voltage, which is defined as the voltage applied to produce a silicon surface potential of zero. The flat-band voltage of EIS is calculated as^27^:

$${{\rm{V}}}_{{\rm{FB}}}={{\rm{E}}}_{{\rm{ref}}}\,-\,{{\rm{\Phi }}}^{{\rm{Si}}}\,{-{\rm{\Psi }}}_{0}\,-\,{{\rm{Q}}}_{{\rm{i}}}/{{\rm{C}}}_{{\rm{i}}}+{{\rm{\chi }}}^{{\rm{Sol}}}+{{\rm{\delta }}}_{{\rm{\chi }}}$$

where E_ref_ is the reference electrode potential with respect to vacuum;$\,\frac{1}{q}$ф^Si^ is the work function of Si, which is equal to 4.7 V; Ψ_0_ is the potential drop in the electrolyte at the insulator-electrolyte interface; C_i_ and Q_i_ are the insulator capacitance and effective charge per unit area, respectively, χ^Sol^ is the surface dipole potential of the solvent; and δ_χ_ is the number of variations of χ potentials. The surface potential Ψ_0_ changes by changing the pH of the solutions, which causes a change in flat-band voltage at the different pHs. The surface potential Ψ_0_ is calculated as^25,28^:

$${{\rm{\Psi }}}_{0}=2.3\frac{kT}{q}({\rm{pHpzc}}-{\rm{pH}})\frac{\beta }{\beta +1}$$

where pH_pzc_ is the value of pH for which the oxide surface is electrically neutral and β is the parameter that depends on the sensitivity of the sensing surface. The site-binding model can also be explained using the equation for capacitance, Q = CV, where Q is the surface charge, C is the double layer capacitance at the insulator interface, and V is the surface potential (Ψ_0_). The surface potential can be expressed as:

$${{\rm{\Delta }}{\rm{\Psi }}}_{0}=-2.3\,{\rm{\alpha }}\,\frac{kT}{q}{{\rm{\Delta }}\mathrm{pH}}_{{\rm{bulk}}}$$

where α is the dimension-less sensitivity parameter consisting of the differential double layer capacitance. The ability of the oxide surface to protonate and deprotonate can be represented by the symbol β.

**Figure 5:**
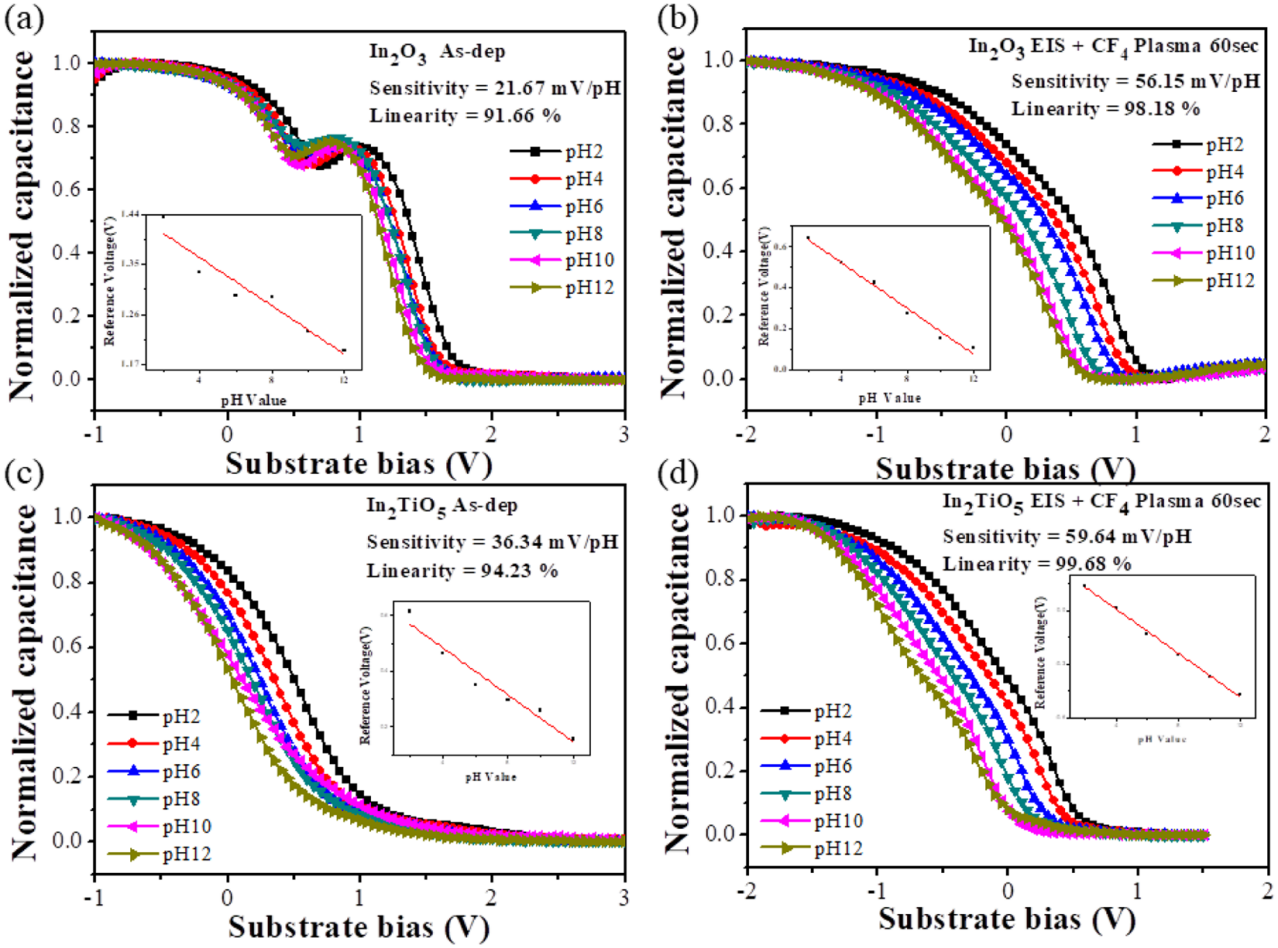
Normalized C-V curve of the sensing membrane of (**a**) as-deposited In_2_O_3_ sample and (**b**) In_2_O_3_ sample CF_4_ plasma treated for 60 sec, and (**c**) as-deposited In_2_TiO_5_ sample and (**d**) In_2_TiO_5_ sample CF_4_ plasma treated for 60 sec.

Figure 5(a–d) represent the capacitance versus substrate bias (C-V) curves of the In_2_O_3_ and In_2_TiO_5_ sensing membranes with and without CF_4_ plasma treatment. The inset of the figures shows the pH sensing properties of the films extracted from the C-V curves. The as-deposited In_2_O_3_ sample displayed a very low sensitivity of 21.67 mV/pH and linearity of 91.66%. The In_2_O_3_ sample treated with CF_4_ plasma for 15 sec displayed higher sensitivity and linearity of 47.03 mV/pH and 98.76%, respectively. The sensitivity and linearity increased to 52.43 mV/pH and 98.83% for samples treated for 30 sec. When samples were treated for 60 sec, the sensitivity and linearity further increased to 56.15 mV/pH and 98.18%, respectively. The as-deposited In_2_TiO_5_ sample displayed a decent sensitivity of 36.34 mV/pH and linearity of 97.65%. For CF_4_ plasma treatments of 15 and 30 sec, the sensitivity and linearity increased to 49.98 mV/pH and 94.23% at 15 sec, respectively, and 55.06 mV/pH and 99.67% at 30 sec, respectively. With a 60-sec CF_4_ plasma treatment, In_2_TiO_5_ samples displayed highest sensitivity and linearity values of 59.64 mV/pH and 99.68%, respectively. The CF_4_ plasma treatment increased the overall sensing surface area by increasing the surface roughness. Thus, the larger sensing surface area will lead to higher sensitivity with more surface sites being present and capable of binding ions present in the electrolyte solution^18^. Therefore, pH sensing membrane treated with CF_4_ plasma for 60 sec can possess high sensitivity and excellent linearity. These properties can be attributed to the formation of a greater number of fluorinated bonds (F-In bonds) on the In_2_O_3_ and In_2_TiO_5_ film surface due to CF_4_ plasma treatment. The deviation in surface potential of the sensing surface after the surface is dipped into solutions with different pH values was determined by the C-V shift. H^+^ and OH^−^attachment at the corresponding binding sites at the sensing surface can alter the surface potential^29^. The present results demonstrate that Ti doping increases the numbers of binding sites on the sensing surface. The grain size becomes larger with higher surface roughness due to incorporation of Ti. Correspondingly the number of binding sites increases with the larger surface area^23^. Therefore, the sensitivity and linearity of In_2_TiO_5_ membranes that are plasma-treated for 60 sec is much higher than those aspects of In_2_O_3_ membranes.

**Figure 6:**
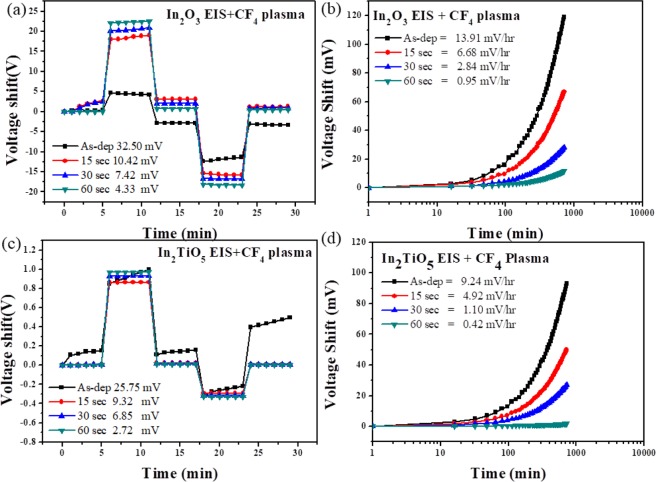
(**a,c**) Hysteresis of In_2_O_3_ and In_2_TiO_5_ sensing membranes for the as-deposited and samples with CF_4_ plasma treatment of various times during the pH loop of 7 → 4 → 7 → 10 → 7 over a period of 30 min. (**b,d**) Drift rate of In_2_O_3_ and In_2_TiO_5_ sensing membranes for the as-deposited and samples with CF_4_ plasma treatment for various times in pH 7 buffer solution.

To investigate the hysteresis effects of the membrane, the samples were immersed in buffer solutions with different pH values in an alternating cycle (pH 7 → 4 → 7 → 10 → 7). Moreover, to test the long-term reliability of the devices, the drift effect of the In_2_O_3_ with CF_4_ plasma treated sensing membrane was measured by a C-V curve in a pH 7 buffer solution for 12 h. The hysteresis voltage and drift rate under the different CF_4_ plasma treatment conditions reveal that the 60-sec In_2_O_3_ film plasma treatment produced the lowest hysteresis voltage and lowest drift rate (4.33 mV and 0.95 mV/h, respectively (Fig. 6(a–d)). In_2_TiO_5_ CF_4_ plasma-treated sensing membrane displayed a hysteresis voltage 25.75, 9.32, 6.85 and 2.72 mV in the as-deposited condition and following 15, 30, and 60 sec of annealing, respectively. Figure 6(d) shows the drift rate for In_2_TiO_5_ devices with CF_4_ plasma treatment. The drift rate of the as-deposited sample was 9.23 mV/h, whereas drift rates of 4.92, 1.09, and 0.42 mV/h were obtained following annealing for 15, 30, and 60 sec, respectively. This was likely because fluorine compensated for the dangling bonds and replaced weaker bonds in the grain boundaries. Thus, the CF_4_ plasma treatment can reduce the trap states in the oxide/silicon interface and improve the hysteresis and drift rate characteristics of the films^18^. The dangling bonds are negated by Ti ions and the defects located underneath of the dielectric surface are compensated^16,23^.

**Figure 7:**
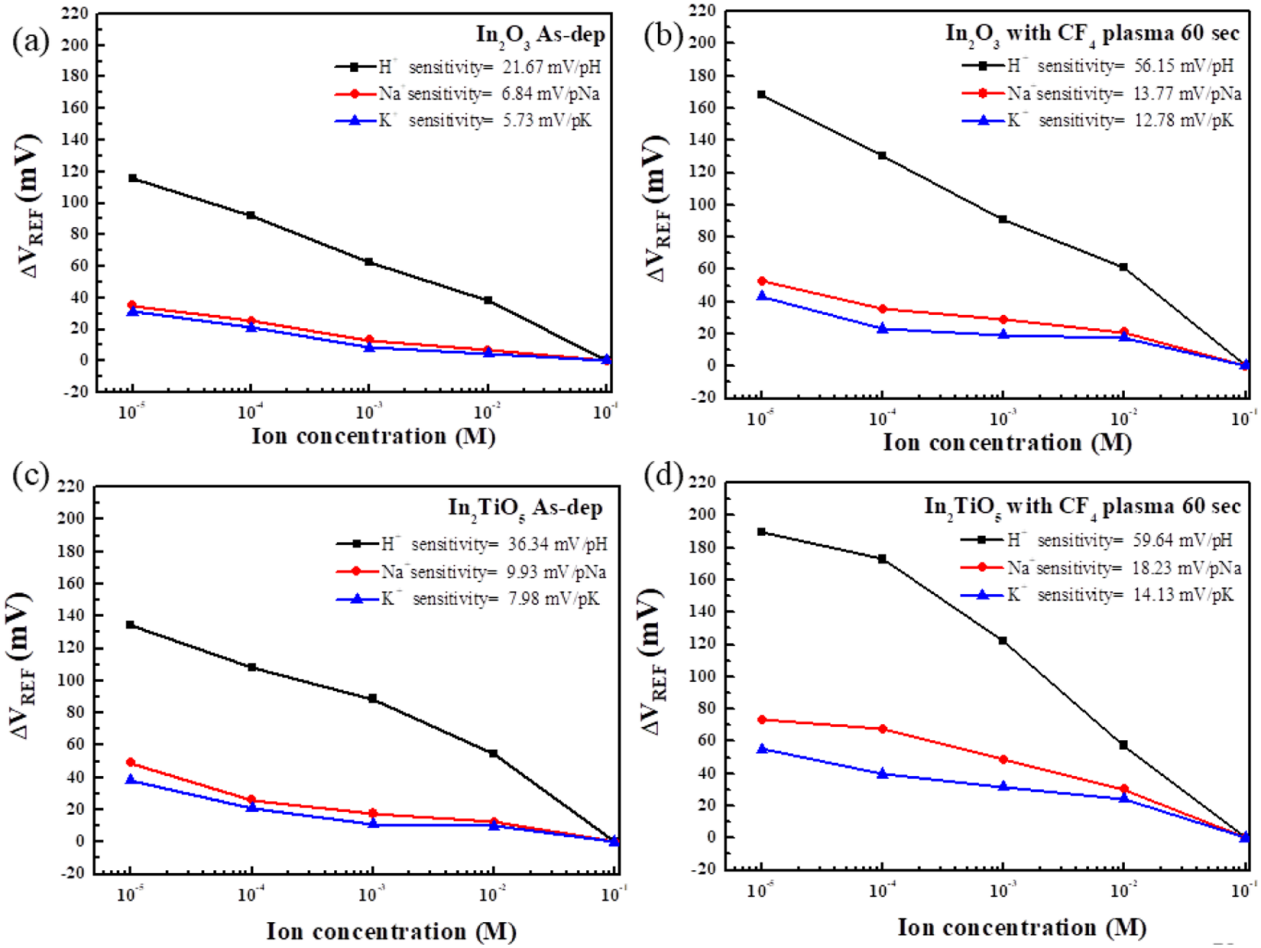
H^+^, Na^+^, and K^+^ sensing properties of as-deposited In_2_O_3_ and In_2_TiO_5_ membrane ((**a,c**) respectively) and In_2_O_3_ and In_2_TiO_5_ membrane ((**b,d**), respectively) treated with CF_4_ plasma for 60 sec.

To analyse the sensing performance of our sensing membrane in the presence of potassium and sodium, we prepared a solution using a micropipette to control the concentrations of sodium and potassium ions ranging between 10^−5^ and 10^−1^ M by injecting 1 M NaCl/Tris-HCl and 1 M KCl/Tris-HCl into buffer electrolyte. Comparisons of H^+^, Na^+^, and K^+^ sensing performances of the as-deposited In_2_O_3_ and In_2_TiO_5_ membrane, and the membrane treated with CF_4_ plasma for 60 sec are presented in Fig. 7(a–d). In_2_TiO_5_ sample treated with CF_4_ plasma for 60 sec had best sensitivities (18.23 mV/pNa and 14.13 mV/pK) compared to the In_2_TiO_5_ as-deposited sample (9.93 mV/pNa and 7.98 mV/pK) and 60-sec CF_4_ plasma treated In_2_O_3_ sample (13.77 mV/pNa and 12.78 mV/pK). For sodium and potassium sensing, considerably lower sensitivity was obtained compared to H^+^ sensing. This can be explained by the heavier and larger Na^+^ and K^+^ ions compared to H^+^ ions.

We also measured the essential human biochemistry data of glucose and urea. They were detected by incorporating appropriate enzymes on the sensing oxide surface. The chemical equations for the reactions that can be used for glucose detection are^30,31^:

$${\rm{\beta }}\,\mbox{--}\,{\rm{D}}\,\mbox{--}\,{\rm{Glucose}}+{{\rm{O}}}_{2}+{{\rm{H}}}_{2}{\rm{O}}\,\mathop{\longrightarrow }\limits^{glu\,\cos \,eoxidase}\,{\rm{D}}\mbox{--}{\rm{glucose}}\,\mbox{--}\,{\rm{\delta }}\,\mbox{--}\,{\rm{lactone}}+{{\rm{H}}}_{2}{{\rm{O}}}_{2}$$

$${\rm{D}}\,\mbox{--}\,{\rm{glucose}}\,\mbox{--}\,{\rm{\delta }}\,\mbox{--}\,{\rm{lactone}}\to \,{\rm{D}}\,\mbox{--}\,{\rm{gluconoate}}+{{\rm{H}}}^{+}$$

The chemical equation for the reactions that can be used for urea detection is^30,31^:

$${{\rm{NH}}}_{2}{{\rm{CONH}}}_{2}+3{{\rm{H}}}_{2}0\,\mathop{\longrightarrow }\limits^{urease}\,2{{{\rm{NH}}}_{4}}^{+}+{{\rm{OH}}}^{-}+{{{\rm{HCO}}}_{3}}^{-}$$

**Figure 8:**
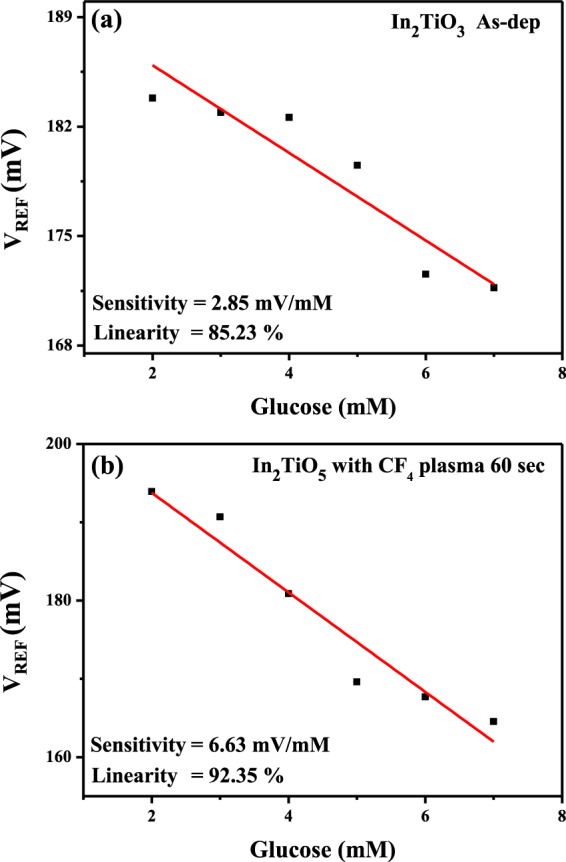
Glucose sensing properties of (**a**) as-deposited In_2_TiO_5_ membrane and (**b**) membrane treated with CF_4_ plasma for 60 sec.

The glucose sensing properties of the In_2_TiO_5_ sensing membrane on the EIS structure with and without the 60-sec CF_4_ plasma treatment are presented in Fig. 8(a,b). The glucose concentration was controlled over the range of 2 to 7 mM. The as-deposited In_2_TiO_5_ sensing membrane exhibited a low glucose sensitivity of 2.85 mV/mM and linearity of 85.23%. After Ti doping and 60-sec CF_4_ plasma treatment, the sensitivity increased to 6.63 mV/mM and the linearity increased to 92.35%.

**Figure 9:**
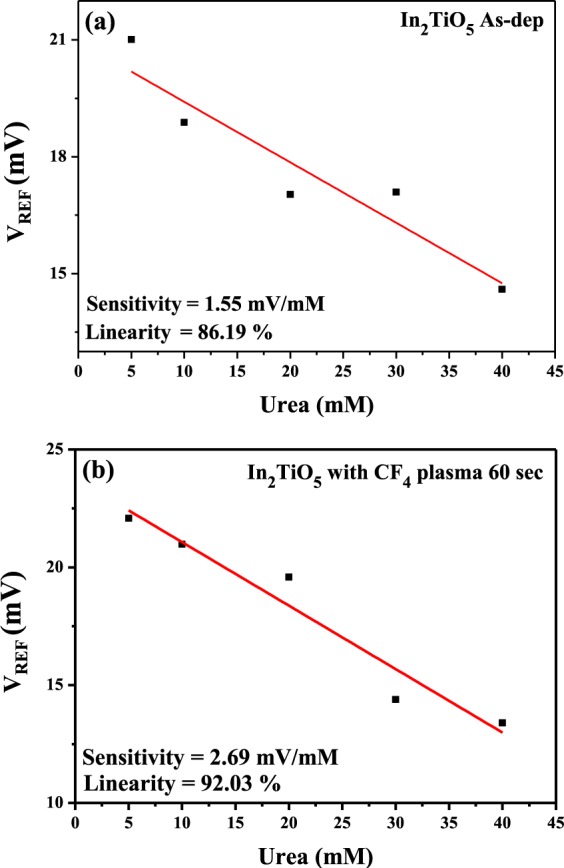
Urea sensing properties of (**a**) as-deposited In_2_TiO_5_ membrane and (**b**) membrane treated with CF_4_ plasma for 60 sec.

The urea concentration was controlled in a range between 5 and 40 mM (Fig. 9(a,b)). The sensitivity values of the In_2_TiO_5_ sensing membrane above as-deposited and CF_4_ plasma treatment in samples treated for 60 sec were 1.55 and 2.69 mV/mM, respectively. Therefore, the 60-sec CF_4_ plasma treated In_2_TiO_5_ sensing membrane has better sensitivity and linearity for the detection of urea. These results indicate that the CF_4_ plasma treatment can increase the interior grain size and increase the number of surface sites, resulting in better sensing performance for the detection of H^+^, Na^+^, K^+^, glucose, and urea.

The test sample of commercially available glucose devices, such as the ACCU-CHECK glucometer, uses a precious metal (Ag/AgCl)^32^. In this study, we used In and Ti as the sensing film. Their use can reduce the purchase price and enable reuse of material. These advantages also include a sensing membrane with high glucose sensitivity and the capability to accurately and sensitively measure other molecules.

Table 1 provides comparative data of the sensing parameters of drift rate, pH sensitivity, hysteresis voltage, glucose, and urea sensing for different EIS devices with TbY_x_O_y_^33^, CeO_2_^34^, ZnO^35^, Sm_2_O_3_^36^, Ti-doped ZnO^37^, and CeO with CF_4_ plasma treatment^38^. The pH sensitivity, hysteresis, drift rate, glucose and urea sensing of the EIS device prepared with In_2_TiO_5_ and incorporating a CF_4_ plasma sensing membrane was superior. CF_4_ incorporated into In_2_TiO_5_ film can enhance the In-O bonding at the In_2_TiO_5_ sensing membrane, which improves the sensing characteristics.

**Table 1:** Comparative data of the obtained sensing parameters of drift rate, pH sensitivity, hysteresis voltage, glucose, and urea sensing for different EIS devices.

Sensing membrane	pH sensitivity (mV/pH)	Hysteresis voltage (mV)	Drift rate (mV/h)	Glucose	Urea	References
TbY_x_O_y_	59.79	5.00	0.26	4.81	—	^33^
CeO_2_	57.60	8.43	1.21	4.74	2.49	^34^
ZnO	42.54	7.37	1.78	3.14	1.81	^35^
Sm_2_O_3_	56.20	6.20	1.29		2.45	^36^
Ti-ZnO	57.56	2.79	0.29	6.42	1.4~3.62	^37^
**CeO with**						
CF_4_ plasma	53.38	—	—	5.83	2.30	^38^
In_2_TiO_5_ with CF_4_ plasma	59.64	2.72	0.42	6.63	2.69	—

## Conclusion

A CF_4_ plasma treated In_2_TiO_5_-based EIS sensor was fabricated for H^+^, Na^+^, K^+^, and glucose sensing applications. The effect of the post-deposition CF_4_ plasma treatment on the physical and electrical characteristics of the sensors was studied. The In_2_TiO_5_ EIS sensor with a 60-sec post-deposition plasma treatment exhibited good sensitivity of 59.64 mV/pH and linearity of 99.68%. Post-deposition CF_4_ plasma treatment improves the crystalline structure and repairs the dangling bonds at the grain boundaries. Furthermore, the 60-sec plasma treatment produced the best material and electrical properties, and achieved ideal sensing capabilities, likely due to defect passivation by fluorine. Furthermore, the Ti-doped In_2_O_3_-based EIS sensor treated with CF_4_ plasma was more responsive to H^+^ compared to Na^+^ and K^+^. The In_2_TiO_5_ sensing membrane with 60-sec CF_4_ plasma treatment also had better sensitivity and linearity than the as-deposited sample for glucose and urea detection.

## Methods

The EIS incorporated In_2_O_3_ and In_2_TiO_5_ sensing membranes were fabricated on 4-inch n-type (100) silicon wafers with a resistivity of 5–10 Ω-cm. After a standard RCA cleaning process, the samples were dipped into a 1% hydrofluoric acid solution to remove native oxide from the surface. A 50 nm-thick SiO_2_ substrate was thermally oxidized on the silicon wafer. Then, (a) a 50 nm-thick In_2_O_3_ film was deposited on the SiO_2_/Si substrate by reactive radio frequency sputtering. (b) Both In_2_O_3_ and Ti were used to co-sputter In_2_TiO_5_ film on SiO_2_/Si stacks in a diluted argon flow (Ar/O_2_ = 20 sccm/5 sccm) with a radio frequency power and process pressure of 100 W and 20 mTorr, respectively. After deposition, the In_2_O_3_ and the In_2_TiO_5_ films were subjected to a post-deposition CF_4_ plasma treatment in a plasma-enhanced chemical vapor deposition system with a radio frequency power of 30 W and a pressure of 500 mTorr for 15, 30, and 60 sec. The back-contact was made by depositing a 300 nm-thick aluminium film on the back side of the Si wafer. The sensing area of the deposited In_2_O_3_ films was defined by an automatic robot dispenser with an adhesive silicone gel to build the final EIS structure on the printed circuit board using a silver gel to form conductive lines. An epoxy package was used to separate the EIS structure and the copper line. The detailed process flow of the EIS structure fabrication is shown in Fig. 1.

Morphological analyses of the In_2_O_3_ and In_2_TiO_5_ sensing membranes included XRD, AFM, and SIMS. These analyses were done to investigate the link between structural and electrical characteristics properties. SIMS instruments use an internally yielded beam of either positive or negative ions focused on a sample surface to generate secondary ions. The generated ions are then transferred into a mass spectrometer across a high electrostatic potential. The depth profiling of elemental and molecular species, as well as isotopic ratios of compounds was evaluated by SIMS analysis. The surface morphologies of the In_2_O_3_ and In_2_TiO_5_ nano-layers were observed using AFM in Bruker Dimension Icon modes with intermittent contact using a silicon tip with a 10 pN/nm spring constant. A sample area of 3 × 3 μm was scanned with actuation rates up to 8 kHz in air and fluid. For XRD analysis of the samples, grazing incidence of X-ray beam CuKa (k = 1.542 Å) radiation was used with an incidence angle step of 0.5° in the diffraction angle range (2θ) from 20° to 60°.
